# Factors Associated with Modern Contraceptive Use Among Out of School Adolescent Girls in Majengo and Njoro Wards of Moshi Municipality, Tanzania

**DOI:** 10.24248/eahrj.v7i1.706

**Published:** 2023-07-12

**Authors:** William Nkenguye, Hunaina Ismail, Ellyagape P. Urassa, Nateiya M Yongolo, Sophia Kagoye, Sia E. Msuya

**Affiliations:** a Kilimanjaro Christian Medical University College (KCMUCo), Moshi, Tanzania; b Kilimanjaro Clinical Research Institute (KCRI), Moshi, Tanzania; c Institute of Public Health, Department of Epidemiology & Biostatistics, Kilimanjaro Christian Medical University College (KCMUCo), Moshi, Tanzania; d Department of Community Health, KCMC Hospital, Moshi, Tanzania; e Institute of Public Health, Department of Community Health, Kilimanjaro Christian Medical University College (KCMUCo), Moshi, Tanzania.

## Abstract

**Background:**

Low uptake of modern contraceptives among adolescents remains a serious public health concern with over 20 million adolescents in need of modern contraceptives are not using any. In Tanzania where the adolescent fertility rate is 112 per 1000, only 15.2% of adolescents are using modern contraceptives. Contraceptive use stands out to be one of the key interventions to reduce the burden of adolescent pregnancy which is high in the country at 22%. There is little information on factors associated with modern contraceptive use among out of school adolescents, who are at an increased risk of adolescent pregnancies.

**Objective:**

To determine the prevalence and factors associated with modern contraceptive use among out of school adolescent girls in Moshi municipality, Kilimanjaro region.

**Methodology:**

This was a population based cross-sectional study, conducted in Moshi municipality in Kilimanjaro region 2 wards; Majengo and Njoro. The wards were randomly selected out of the 21 wards in the region. Household survey was conducted in the wards and adolescents aged 10 to 19 years who were out of school and consented and or assented to participate in the study were recruited. Data was collected using Kobo Collect ^TM^ on an Android device. Data was analysed using SPSS version 20. To determine the factors associated with modern contraceptive use, crude and adjusted analysis using logistic regression analysis was done.

**Results:**

A total of 298 out-of-school adolescents were enrolled, with a median age of 19 (IQR 17-19) years. The prevalence of ever use of modern contraceptives among 154 sexually active adolescents was 51%, and 35% were current users of the methods. Two of common methods ever used were; injectables (27.3%) and male condoms (3.2%) respectively. Factors independently associated with ever use of modern contraceptives were; being married or cohabiting (aOR: 5.7) and having 2 or more sexual partners in the past 12 months (aOR: 5.9).

**Conclusion:**

Ever and current use of modern contraceptives among out-of-school adolescents were reported at 51% and 35% respectively. Respondent's marital status and number of sexual partners was associated with ever use of modern methods. Strengthening of adolescent-friendly SRH services outside facility setting is needed given very few are currently using a modern method. Further, through inter-sectoral collaboration interventions to keep adolescent girls at school should be strengthened.

## INTRODUCTION

The World Health Organization (WHO) defines adolescents as young people between 10 and 19 years of age.^1^ Uptake of modern contraceptives among adolescents is low globally.^2^ Globally, over 20 million adolescents in need of modern contraceptives are not using any.^2^ In Tanzania, 96% of adolescents are aware of modern contraceptives but only 15.2% use any of these methods.^3^ As a result, an estimated 11% of births globally occur among girls aged 15 to 19 years, 95% of which occur in Low- and Middle-Income Countries (LMICs).^4^

Numerous studies report that the overall pooled prevalence of pregnancy among adolescents is 19.3% in sub-Saharan Africa (SSA), and that 120 out of every 1000 girls within SSA experience unplanned pregnancies. This prevalence is reported highest in the East African region at 21.5%.^5^

Tanzania has the 17^th^ highest adolescent fertility rate in Africa with a fertility rate of 81 births per 1,000 women aged 15 to 49 and 112 per 1000 adolescents aged 15 to 19 years.^6^ Adolescent (aged 15-19) pregnancy rates have increased in Tanzania since the last 2010 Demographic and Health Survey (DHS) from 23% to 27% in 2015/16 and then slightly decreased to 22% in 2022.^7^ Tanzania also has a high teenage pregnancy rate with at least 6% of teenage girls aged 15 to 19 years currently being pregnant.^8^ Moreover, the current Modern Contraceptive Prevalence Rate (m-CPR) among adolescents is 15.2%. This shows a significant decline from 18.9% in 2015 and 2016 which is significantly lower compared to the national average of 32%.^9^ So unmet needs for Family Planning is high among adolescents aged 15 to 19 years in Tanzania and there is poor progress in rates of unmet need for modern contraception use (30% in 2004/05, 25.3% in 2010 and 26.5% in 2015/16 respectively.^8^)

Stemming from these concerns, adolescent pregnancy results in poor health and socio-economic consequences not only to the adolescent mother but also on their babies.^10^ Poor health outcomes such as increased risk of eclampsia, puerperal endometritis and systemic infections due to abortion may all result in increased maternal morbidities and mortality.^11^ Also, babies born to adolescent mothers have a higher risk of low-birth-weight preterm delivery and severe neonatal conditions.^12^ Moreover, adolescent pregnancies cause poor socio-economic outcomes to adolescent girls due to stigma, rejection or violence by the partner, parents or peers which further results into increase in the number of school dropouts. From UNFPA (United Nations Population Fund), Tanzania Family planning fact sheet, it is reported that, between 2003 and 2011, 55,000 girls dropped out of school in Tanzania because they were pregnant, this will definitely impact their future education and employment opportunities.^7^ About 1.5 million out of 9.9 million adolescents in Tanzania are out of school and 40% of these never attended secondary education.^13^

Modern methods of contraception include; sterilisation, Intrauterine Devices (IUDs), subdermal implants, oral contraceptive pills, condoms and other barrier methods, injectable, emergency contraceptive pills, contraceptive patches, spermicidal foams and other agents and vaginal ring.^14^ Modern contraceptives have the potential to prevent adolescents from conceiving and thus prevent negative health consequences to the adolescent mothers and their babies, as well as socio-economic consequences such as dropping out of school, stigma and violence.^15^

According to TDHS 2015/2016, 86.7% of married adolescent girls and 66.9% of unmarried sexually active adolescent girls do not use modern contraceptive methods, and in a 2022 report, 84.8% of unmarried sexually active adolescent girls do not use modern contraceptive methods. In Kilimanjaro region, prevalence of modern contraceptive use among women of reproductive age is 48.9%, however, there is a dearth of information on the use of contraceptive among adolescent girls.^16^

Several interventions have been put in place by the Government of Tanzania and stakeholders in order to increase modern contraceptives uptake among adolescent girls. A 5-year Implementation Plan for Family Planning 2018-2022 was strategised for Tanzania mainland by the Ministry of Health, Community Development, Gender, Elderly and Children (MoHCDGEC) of United Nation of Tanzania; Plan sets targets for increased use of all family planning methods. Aims to increase the uptake of family planning methods and identifies priority areas as well as the financial resources required.^7^

Additionally, The Government of Tanzania also updated its commitment at the Family Planning Summit in London, UK on July 11, 2017. Tanzania will increase the availability of modern contraceptive methods at all levels of its health system; specifically, increase its allocation for Family Planning (FP) commodities from 14 billion Tanzania shillings (Tsh) in 2017 to 17 billion Tsh by 2020, Expand the availability of at least 3 modern contraceptive methods at primary level and at least 5 modern contraceptive methods at secondary and tertiary level facilities from 40% to 70% and Scale-up the number of health facilities providing youth-friendly reproductive health services from 30% to 80%.^17^

Interventions for Adolescent Sexual and Reproductive Health (ASRH) using mobile phones have shown positive progress. Findings suggest that mHealth interventions are becoming a more common method to connect youth to Sexual and Reproductive Health (SRH) information and services in LMICs, and evidence is emerging that mobile phones are an effective way to reach young people and to achieve knowledge and behaviour change.^18^

In Kilimanjaro however, there is a dearth of published information on modern contraceptive use specific for out of school adolescents.

Despite the interventions, the burden of adolescent pregnancies still remains regardless of interventions on modern contraceptive use in Tanzania. In Kilimanjaro, 6% of girls became pregnant before the age of 19 years in 2015 and this increased to 7.6% in 2022.^8^ There is still a long way to achieving the target 3.7 of the Sustainable Development Goals (SDGs) which calls on countries; to ensure universal access to sexual and reproductive health-care services, including for Family Planning, information and education, and the integration of reproductive health into National Strategies and Programs by 2030.^19^ Therefore, this study aimed at determining the prevalence and factors associated with modern contraceptive use among out of school adolescent girls in Majengo and Njoro wards in Moshi Municipality, Kilimanjaro region. Being conducted at ward level, a sub national level where policies are implemented in the country, this study is the first to be conducted among out of school adolescents and therefore will give a blueprint that will guide policies targeted at increasing the uptake of modern contraceptives among adolescents.

## METHODS

### Study Design and Setting

This was a household based cross sectional study conducted in August 2020 at Moshi Municipality in Kilimanjaro region. The region is situated in Northern Tanzania and is subdivided into seven districts namely, Moshi municipality, Moshi rural, Rombo, Mwanga, Same, Hai and Siha. The main economic activities are food and cash crop production, commercial activities, tourism and forestry.

The Moshi municipality covers an area of about 59 square kilometres and is the smallest municipality in Tanzania by area. According to the 2018 estimates, Moshi municipality has a population of 225,225 people of which 52.2% are females. The municipality is administratively divided into 21 wards, due to COVID 19 movement restrictions, only 2 wards could be enrolled for the study; Majengo and Njoro and these were randomly selected.

### Study Participants, Sampling and Sample Size

The study involved out of school adolescent girls aged 15 to 19 years from selected wards in Moshi municipality and who were willing to participate. Adolescents who did not consent and/or assent or those who had serious medical conditions that prompted them not to participate in the study were excluded. Moshi municipality was purposely selected out of 7 districts due to COVID 19 movement restrictions. Two wards were randomly selected out of 21 wards in Moshi municipality. Small sheets of paper containing one of the names of the 21 wards in Moshi municipality were put in a jar, vigorously shaken and 2 sheets were randomly drawn from the jar. Majengo and Njoro wards were selected. In each of the selected ward, streets were randomly selected. All the households with adolescents meeting the inclusion criteria were visited and adolescents available were invited to participate. All participants who were available on the day of the visit or agreed to be located where they work and willing to take part in the study were enrolled. Fisher's formula was used to estimate the minimum required sample size with a prevalence of 32.7%, based on a study conducted in Uganda^2^ and adding a 15% of non-response, arriving at a sample size of 303, however due to the COVID 19 pandemic movement restrictions only 298 samples were reached.

### Data Collection

Data collection tool used was a questionnaire which had questions in English and Swahili. The tool had open and closed ended questions and was in an electronic format, kobo collect. The sections in the tool were; socio demographic characteristics, economic status, reproductive status, sexual behaviour, accessibility and availability of contraceptive services and these were filled by the interviewer with help of the data collection officer. During the day of data collection, face to face interviews were also done to collect more information from respondents. Interviewers were trained and emphasised to observe and follow standard protocols to avoid interviewer bias. After the interview, adolescents were given brief information about what is family planning and contraception, where to obtain family planning and contraceptive services. The data collection process was conducted for 4 weeks.

### Study Variables

In this study, the dependent variable was ever use of modern contraceptive which was a binary outcome and coded as, (Yes) for those who had ever used modern contraceptive and (No) for those who had never used any modern contraceptive.

Independent variables included Socio-Demographic Information; Age, Residence, Marital status, Religion, Education level, Age of the partner, Partners education level, currently working status, Sexual and Reproductive Health Information; Age at sexual Debut, Parity, Ever had sexual intercourse, Last sexual intercourse, Number of sexual partners in a lifetime, Modern contraceptive knowledge, Discussion with partner on condom use, Source of modern contraceptives.

### Data Processing and Analysis

At the end of each day, after collection of information, the team passed through the questionnaires to check if they were filled correctly and test for validity. Data from Kobo collect was then exported to Statistical Package for Social Sciences (SPSS) version 20 (IBM Corporation, Armonk, NY, USA). Data was analysed using SPSS version 20 (IBM SPSS Statistics for Windows, Version 20.0. Armonk, NY: IBM Corp). Continuous variables were summarised using measures of central tendency with respective measures of dispersion. Categorical variables were summarised by frequency and percentages. To determine the factors associated with modern contraceptive use, crude and adjusted analysis using logistic regression analysis was done. A magnitude of association was assessed using Odds Ratio (OR) and their respective 95% Confidence Intervals. A *p*-value of <0.05 was considered statistically significant.

### Ethical Consideration

Ethical clearance was obtained from Kilimanjaro Christian Medical University College Research Ethics Committee prior to commencement of the study. The permission letter from the university was taken to the District Medical Officer (DMO) of Moshi municipality to request permission to conduct the study. The DMO gave written permission that requested the ward leaders to assist us in carrying the research project. Ward leaders introduced the researchers to a link person who was an indigenous and well familiar with the households which had out of school adolescent girls in that particular ward. During the day of data collection, in addition to assent from the adolescent girls under 18 years of age, written informed consent was sought for from a parent or guardian or next of keen or employers for all participants and thumb prints were used for those who could not write. For those above 18 years, consent was sought prior to commencement of the study All participants were identified using identification numbers and not names to guarantee anonymity.

## RESULTS

### Background Characteristic of the Study Participants

#### Participant Socio-Demographic Characteristics

A total of 298 out of school adolescent girls participated in this study. The median age was 18 years (IQR 17-19). Majority of the study participants (79.6%) lived with their parents or guardians and were single (75.8%). More than half of the participants (58%) dropped out of school during their secondary education level. (Table 1)

**TABLE 1:** Participant Socio-Demographic Characteristics (N=298)

Variable	Frequency	Percentage
Age		
10–14	28	9.2
15–19	270	90.6
Residence		
Urban	295	99.0
Rural	3	1.0
Marital status		
Single	226	75.8
Married/Cohabiting	69	23.2
Widowed/Divorced/Separated	3	1.0
Education level		
No formal education	13	4.4
Primary education	112	37.6
Secondary education and above	173	58.0
Religion		
Christian	117	39.3
Muslim and others	181	60.7
Living with		
Parent/guardian	237	79.6
Partner/Alone	61	20.4
Alcohol consumption		
Yes	27	9.1
No	271	90.9

#### Participant's Sexual and Reproductive Health Characteristics

Among the 298 participants, 154 (51.7%) reported ever having sexual intercourse. The mean age at sexual debut among the 154 sexually active adolescents was 17 (SD of 1.7) years and 85 (55%) reported to have ever been pregnant, Table 2

**TABLE 2:** Participants Sexual and Reproductive Health Characteristics (N=154)

Variable	Frequency	Percentage
Ever had sexual intercourse (N=298)		
No	144	48.3
Yes	154	51.7
Age at sexual debut		
<15	12	7.8
≥15	142	92.2
Parity (N=85)		
One	70	45.5
Two +	16	10.4
Last sexual intercourse		
3 months ago (N=154)	94	31.5
12 months ago (N=60)	34	11.4
Number of sexual partners in a past 12 months		
Only one	143	92.9
Two or more	11	7.1
Knowledge on modern contraceptives (N=298)		
No	100	33.6
Yes	198	66.4
Ever used modern contraceptive		
No	76	49.4
Yes	78	50.6

### Prevalence, Preference, Availability and Access of Modern Contraceptive Use

The proportion of 154 sexually active adolescent girls who have ever used modern contraceptives was 50.6%. (Table 2). Most common method of contraceptive ever used by the participants was Injectables (27.9%), followed by hormonal pills (3.2%), male condoms (3.2%) and Implants (2.6%). 82.2% of adolescents admitted that it was difficulty to get modern contraceptives and the commonest source of modern contraceptives reported were health facilities (80.3%). Majority of participants (49%) did not know whether or not Modern contraceptives are offered for free. Media (Television and radios) positively impacted awareness of modern contraceptives as reported by approximately 30% of the respondents, Table 3.

**TABLE 3:** Preference, Availability and Access of Modern Contraceptives among Out of School Adolescent Girls in Majengo and Njoro Wards, Moshi Municipality

Variable	Frequency	Percentage
Modern method Currently used (N=154)		
Injectables/Depo Provera	43	27.9
Hormonal pills (Oral)	5	3.2
Male condoms	5	3.2
Implants	4	2.6
Methods of Contraception Known (N=154)		
Injectables/Depo Provera	62	40.3
Hormonal Pills (Oral)	57	37.0
Female condoms	16	10.4
Intrauterine device (IUD)	12	7.8
Implants	9	5.8
Female sterilization (BTL)	2	1.3
Vasectomy	2	1.3
Male condoms	0	0.0
Source of Contraceptive (N=81)		
Health Facilities	65	80.3
Pharmacies	23	25.9
From Friends	3	3.7
Are methods offered for Free Or payment (N=298)		
Don't Know	146	49.0
Free	87	29.2
Have to Pay	65	21.8
Is it easy or difficult to get/access contraceptive methods in this area (N=298)		
Difficulty	245	82.2
Easy	53	17.8
Source of information on Contraceptives (N=198)		
Health facility	93	47.0
Friends/peers	85	42.9
Television	59	29.8
Radio	58	29.3
Family member (sister/brother)	35	17.7
Health care workers	28	14.1
Internet	25	12.6
Poster/Banner	21	10.6
Parents	27	9.1
Sexual partner	15	7.6

### Factors Associated with Ever Using Modern Contraceptives Among Sexually Active Out of School Adolescent Girls

On crude analysis, age, marital status and knowledge on modern contraceptives were the factors associated with ever use of modern contraceptives. The prevalence of ever using modern contraceptives was observed to increase with age, was higher among married/cohabiting adolescent girls and those with knowledge of modern contraceptive. (Table 4).

**TABLE 4:** Factors Associated with Ever Use of Modern Contraceptives Among Sexually Active Out of School Adolescent Girls

Factors	Ever used m-CRP n (%)	Crude Analysis			Adjusted Analysis		
		Odds Ratio	95% Cl	p-value	Odds Ratio	95% Cl	p-value
Age		1.57	1.06, 2.33	0.023	1.50	0.95, 2.35	0.079
Residence							
Urban	77 (50.7)	1		0.985	1		
Rural	1 (50.0)	0.97	0.06, 15.86	0.985	1.41	0.04, 56.29	0.855
Marital status							
Single	29 (34.1)	1			1		
Married/Cohabiting	48 (72.7)	5.15	2.55, 10.40	<0.001	5.66	2.63, 12.12	<0.001
Widowed/Divorced/Separated Education level	1 (33.3)	0.97	0.08, 11.10	0.978	0.37	0.02, 5.94	0.485
No formal education	3 (100)	1					
Primary education	27 (58.7)	1.69	0.84, 3.40	0.144			
Secondary education and above Religion	48 (45.7)	-	-	-			
Christian	23 (43.4)	1			1		
Muslim & Other	55 (54.5)	1.56	0.80, 3.05	0.193	1.41	0.65, 3.06	0.391
Age at sexual debut							
<15	7 (53.8)	1					
≥15	71 (50.4)	0.87	0.28, 2.72	0.810			
Parity							
Only one	50 (71.4)	1					
Two +	12 (80)	1.6	0.41, 6.28	0.500			
Number of sexual partners in a past 12 months							
Only one	61 (64.9)	1			1		
Two or more	11 (32.4)	2.78	0.71, 10.91	0.142	5.85	1.33, 25.63	0.019
Modern contraceptive knowledge							
No	70 (49.0)	1	1				
Yes	8 (72.7)	4.18	1.31, 13.34	0.016	3.54	0.91, 13.79	0.068

On adjusted analysis while controlling for other factors, the factors that were significantly associated with ever use of modern contraceptives were; being married/cohabiting and having 2 or more sexual partners in the last 12 months. Those who were married or cohabiting had significant 5.66 higher odds of using modern contraceptives compared to those who were single (OR: 5.66; 95% CI: 2.63, 12.12). Also, those who had 2 and more sexual partner in the last 12 months had significant 5.85 higher odds of ever use of modern contraceptives compared those who had only one sexual partner (OR: 5.85; 95% CI: 1.33, 25.63).

## DISCUSSION

The aim of this study was to determine the prevalence and factors associated with modern contraceptive use among out of school adolescent girls in Moshi Municipality, Kilimanjaro region. In this study, the prevalence of ever using modern contraceptives was 50.6%, prevalence in this study is high when compared to a study that was conducted in Ghana in which the prevalence was reported to be 18.3%.^20^ A similar study conducted among countries in SSA also reported a lower prevalence of 21.1%.^21^ Similarly, the findings of this study were slightly higher than findings of related study done in Tanzania in which the prevalence of contraceptive use among adolescents who had ever had sex was reported to be 43.6%.^22^ The slight difference in the results could be due to the small proportion of participants in this study who had ever had sex (154) compared to this study's 260. This can also be explained by the interventions done by the government, such as; introduction to family planning services that are free and adolescent friendly tailored programs aimed at meeting the demands of adolescents' increase in knowledge, skills and use of interventions.^23^ A study in Ethiopia reported a prevalence of 39.6% and the odds of contraceptive use were lower among those with no formal education which is similar to our study's observation.^24^ Similar findings were observed in a study conducted in South Africa and Zambia in which adolescent with formal education were more likely to use contraceptive.^25,26^

Participants who reported being married or cohabiting had higher odds of modern contraceptive use compared to those who reported to be single. These findings align with findings of a study done in Ghana in which contraceptive use was high among married female adolescents.^20^ A similar study done among countries in the SSA reported similar findings.^21^ Married female adolescents are more likely to use modern contraceptive when compared to single female adolescents probably because these maybe more capable to afford contraceptive as they have partner support. Also, married female adolescents are more likely to engage in sexual activity than single female adolescents.

Number of sexual partners in the last 12 months was also a significant factor associated with modern contraceptive use. Adolescent girls who had 2 or more sexual partner were 5.85 times significantly more likely to use modern contraceptive when compared to single adolescent as demonstrated a similar study that was conducted in Nigeria.^27^ This might be as a result of increase in sexual engagement. Nevertheless, Adolescents with 2 or more children had higher odds of using contraceptive methods compared to those with one or no child, these findings align with a study conducted in Zimbabwe.^28^

## CONCLUSION

Sexual and reproductive health is an essential, sensitive matter especially when targeting modern contraceptive use which has a huge role to play when addressing adolescent pregnancy as addressed in Sustainable development Goals target 3.7. This study reports a prevalence of modern contraceptive use of 50.6% among those who have ever had sex. The study, also reports that most of the participants had modern contraceptive knowledge. Furthermore, we report that participants who used modern contraceptives were mostly among those who dropped out of primary education. The study also notes that; age, marital status and knowledge on modern contraceptive are significant predictors of use of modern contraceptive.

Therefore, The government should strengthen inter-sectoral collaborations and interventions that spread awareness in the community about modern contraceptive use, especially among out of school adolescents.

### Strengths and limitations of the study

#### Strengths

This was a house-hold based survey where participants were selected randomly. This enabled us to capture and observe a range of issues concerning modern contraceptives and helped us to identify how people conceptualise their situation, how they interrelate socially, and how they modify their beliefs. The participants got a chance to individually address their concerns on modern contraceptive use after the interview.

The study was conducted at a subnational level, that is the level of wards. Since all health-related policies are implemented at a subnational level, finding of this study therefore, give a spectrum of what is happening at a subnational level and thus lays a blueprint in creation of policies on contraceptive use among out of school adolescent girls.

#### Limitations

The study was conducted during the COVID 19 pandemic. Travel restriction limited the number of districts that could be enrolled for the study. Out of 7 districts, only one district was considered, and only 2 wards were enrolled out of the 21 wards in Moshi Municipality.

During data collection, questions on sexuality were a very sensitive matter to ask girls below the age of 15 years, and thus some of the participants could have under-reported their sexual activity due to social desirability bias. Some parents preferred that their daughters are interviewed in their presence. This might have interfered with the participants' freedom to freely respond to asked questions, and thus might have an effect on the reported findings.
